# Six-year follow-up of an abstinence-based, food addiction recovery approach to weight management

**DOI:** 10.3389/fpsyt.2025.1584201

**Published:** 2025-06-02

**Authors:** Susan Peirce Thompson, Nadia M. Briones, Aaron Blumkin, Betty A. Rabinowitz

**Affiliations:** ^1^ Department of Brain and Cognitive Sciences, University of Rochester, Rochester, NY, United States; ^2^ Bright Line Eating Solutions, LLC, Rochester, NY, United States; ^3^ School of Medicine, New York Medical College, Valhalla, NY, United States; ^4^ Consultant, Rochester, NY, United States; ^5^ Department of Medicine, University of Rochester Medical Center, Rochester, NY, United States

**Keywords:** food addiction, ultra-processed food addiction, obesity, weight loss, weight loss maintenance, abstinence-based treatment, dietary compliance, bright line eating

## Abstract

**Introduction:**

Research shows that the average person has a slightly addictive relationship with food, manifesting two or more symptoms of food addiction, and more than one in eight people have clinically diagnosable food addiction. Meanwhile, obesity and food addiction share some neurological mechanisms and are correlated in the general population. Could an abstinence-based approach to food addiction recovery be a viable long-term weight loss or weight maintenance strategy?

**Methods:**

The current study presents six-year retrospective follow-up data from a cohort of participants who started an abstinence-based food addiction recovery program for weight loss in October of 2017. Survey responses from 267 participants were analyzed and compared to baseline self-reported data from six years prior.

**Results:**

At six years, 71.8% of participants were maintaining greater than 5% weight loss. There was a statistically significant association between sustained weight loss and both current program membership (p<0.001) and degree of adherence to the abstinence-based food plan (p<0.001). Adherence was associated with weight loss outcomes in a dose-response manner. The average sustained weight loss for current members who followed the program was 13.9%.

**Discussion:**

In spite of the methodological challenges with this type of study, the results do suggest the long-term efficacy of a food addiction recovery approach to weight loss. They also help validate the notion that food addiction may be a significant contributor to the multi-factorial etiology of obesity and indicate the need for further research into the viability of abstinence-based food plans as tools for weight management.

## 1 Introduction

Estimates of food addiction prevalence in the general population vary across studies, reflecting differences in methodologies and sample characteristics. A 2021 systematic review and meta-analysis of 272 studies, with an erratum published in 2024, found an overall food addiction prevalence in the general population of 24% with a prevalence in non-clinical samples (those without weight or eating disorder comorbidities) of 14%. This review also noted higher rates of food addiction among individuals with eating disorders (55%-84%) and weight-related comorbidities (30-31%) (1, 2). Another study from the University of Michigan, focusing on adults over 50, found that about 13% exhibited signs of food addiction (3). These variations suggest that prevalence rates can differ widely based on factors such as age, assessment tools, and specific population characteristics. Regardless of these variations, food addiction is clearly a common and significant issue in the general population.

Beyond clinically diagnosed food addiction, many individuals exhibit an addictive relationship with food, as evidenced by symptoms such as difficulty controlling intake or repeated failed attempts to cut back. Gearhardt et al. (4) highlighted this phenomenon in their research using the Yale Food Addiction Scale (YFAS) 2.0, identifying an average of 2.38 symptoms per individual on average (4). However, most of these individuals do not experience “clinically significant impairment or distress” disqualifying them from a formal diagnosis of food addiction (5).

Food addiction symptoms likely contribute to widespread struggles with weight management and, specifically, obesity. Research supports the neurobiological overlaps between obesity and addiction. For example, Volkow et al. (2013) highlighted shared neurobiological mechanisms, such as dysregulated dopamine pathways, which contribute to compulsive eating behaviors and weight gain (6). A study published in *PLOS ONE* found that approximately 88.6% of individuals diagnosed with food addiction were classified as overweight or obese based on Body Mass Index (BMI) criteria. Similarly, when using body fat percentage as a measure, 80% of those with food addiction fell into the overweight or obese categories (7). These findings suggest that a substantial proportion of individuals exhibiting food addiction symptoms also experience overweight or obesity, highlighting the close relationship between addictive eating behaviors and increased body weight. It is important, though, to emphasize that some individuals who are diagnosed with food addiction have normal weight or are even underweight (8).

Like addiction in general, food addiction has two distinct manifestations: substance addiction and process addiction (9). The substance addiction component refers to the addiction to a specific substance or ingredient, such as sugar, flour, or other highly palatable, ultra-processed foods or ingredients. The process addiction component, also called behavioral addiction, refers to the addiction to certain behaviors or processes such as binge eating, compulsive eating, nonstop grazing, and an ongoing inability to refrain from these behaviors. Effective treatment for food addiction must address both the substance and process addiction components.

There are few studies evaluating therapeutic approaches to food addiction and their long-term outcomes, especially programs that address both the substance and process addiction components of food addiction (10).

One notable study evaluated the short-term outcomes of a program using a low-carbohydrate, whole-food abstinence-based approach combined with education and social support to treat symptoms of ultra-processed food addiction (11). The study included 103 self-selected participants from the UK, North America, and Sweden. At 3 months participants reported statistically significant reductions in food addiction symptoms (measured by mYFAS2 and CRAVED scales) as well as modest weight loss (average 2.8% weight loss after 10–14 weeks) and improved mental wellbeing. Weight loss was a dependent variable but not an explicit aim of the intervention.

The current study explores whether an abstinence-based food plan coupled with a comprehensive food addiction recovery program that addresses both the substance and process components of the addiction could provide an effective, long-term alternative to traditional weight management programs in both the general population and among individuals identified as food addicted. The study hopes that by reframing weight management through the lens of addiction, more sustainable and impactful interventions can be developed for individuals struggling with obesity.

Six-year retrospective follow-up data are presented from a cohort of 276 participants who started an abstinence-based program grounded in a food addiction recovery framework in the fall of 2017. The study evaluates the long-term self-reported weight loss outcomes of participants six years after they completed the program’s first course (the “Boot Camp”) and identifies the participant and program characteristics most associated with sustained weight loss success.

Findings from this study will contribute to the ongoing discourse on effective weight management strategies, highlighting the potential of addiction-focused interventions. The findings will also shed some light on the broader implications of incorporating addiction frameworks into obesity treatment paradigms.

## 2 Materials and methods

### 2.1 The program

#### 2.1.1 Education

Bright Line Eating (BLE) is a commercial, online educational program with multiple courses beginning with an eight-week “Boot Camp” that teaches participants about the neuroscience of food addiction and how to recover. Most participants invest $497 USD for the Boot Camp, but some receive scholarships. Through approximately 15 short, weekly training videos, participants are taught that sugar and flour create dopamine downregulation in the nucleus accumbens, similar to the effects of other drugs of abuse, and that this causes food cravings. They are also taught that sugar and flour cause leptin resistance by increasing insulin, triglycerides, and inflammation and that leptin resistance leads to excessive hunger and overeating. At the beginning of the program the abstinence-based food plan is explained, including the scientific rationale for it, and over the ensuing eight weeks of video modules, cognitive behavioral strategies for addiction recovery are introduced and positive psychology interventions to increase well-being are taught. The Boot Camp is tailored to the weight loss phase and later courses in the program outline how to succeed in the maintenance phase.

#### 2.1.2 Abstinence

The program is built on four fundamental rules or “bright lines” that create the framework of abstinence and food sobriety. The expectation is for abstinence to continue beyond the Boot Camp to avoid relapse and intermittent reinforcement. There is, however, no penalty for participants struggling with adherence. The first two bright lines address the substance addiction to triggering ingredients while the second two bright lines treat the process addiction to the behavior of eating itself. Thus, abstinence in the program consists of:

1. No Sugar – Abstain entirely from all added sugars (*e.g.*, honey, maple syrup, molasses, agave, stevia, any additive ending in “-ose”, all non-nutritive sweeteners, alcohol, etc.).
2. No Flour – Abstain entirely from all forms of flour (*e.g.*, wheat flour, almond flour, coconut flour, etc.).
3. Eat Only Meals – Eat only at structured mealtimes without grazing or snacking in between.
4. Weigh and Measure Quantities – Use a digital food scale to weigh food according to pre-determined quantities.

#### 2.1.3 The food plan

The food plan consists of five food categories: proteins, vegetables, fruits, grains, and fats. Whole, fresh fruit is included in the plan, but fruit juice and dried fruit are not. The plan consists of three meals per day with a balanced macronutrient profile and a moderate (not extremely low) calorie content. The plan is not benchmarked to calories but rather to food categories and quantities so actual calories consumed vary depending on the specific foods the person selects each day, but average caloric ranges are 1,000-1,500 calories per day for women and 1,300-1,800 calories per day for men. Meals consist of specific quantities of each food and participants have wide discretion to select which food they will eat from tables that list a large number of options for each category. Participants are taught to plan out their specific menus the night before and write down precisely what they will eat the next day. The plan allows for adjustments based on individual needs (*e.g.*, modifications for plant-based preference, food allergies, medical dietary needs, and so forth).

The elimination of sugar, flour, and snacking might suggest this food plan is a form of Therapeutic Carbohydrate Reduction (TCR). However, the presence of whole, fresh fruits; whole grains; and a variety of vegetables including potatoes and other starchy vegetables, results in a daily meal plan with 35-50% carbohydrate content. This is not ketogenic and not necessarily a carbohydrate reduction (depending, of course, on the individual’s prior diet), but it is certainly a dramatic shift from refined to unrefined carbohydrate consumption.

#### 2.1.4 Coaching and community support

In accordance with research that shows that both professional (12) and peer-to-peer support (13) facilitate recovery from addiction, Boot Camp participants have access to weekly live group coaching calls to get personalized help as well as a large online support community in which over one hundred posts and comments are made each day. The online community is moderated by the program staff according to strict Community Guidelines to maintain “a climate of love and support.” Participants are encouraged, but not required, to engage with one or more accountability buddies and to set up a weekly “Mastermind Group” call with three other participants following a script of sharing and feedback to provide mutual support.

### 2.2 The research methodology

#### 2.2.1 Participant recruitment

The participants in this study were initially drawn from the cohort of people who attended the October 2017 Boot Camp, totaling 1,876 individuals. Because we assumed repeated follow-ups by telephone would be necessary to secure participation, initially only those participants for whom we had phone numbers were deemed eligible for participation. 524 participants met this criterion (27.9%).

These 524 participants were invited to participate in the research study via an initial email. Two follow-up emails were sent to those participants who had not opened the initial email. Once a participant completed the survey, they no longer received any additional communication. There were 215 survey responses from this email effort, representing a 41% response rate.

One week after the last email, the 309 non-responders (59%) were contacted via telephone or text message an additional three times at weekly intervals to solicit their participation. Of the 309 remaining people, 276 were reached via phone or text message (89.3%), and 33 could not be reached (10.7%). These efforts yielded another 101 completed surveys representing an aggregate 60.3% response rate to this stage of data collection.

**Figure 1:**
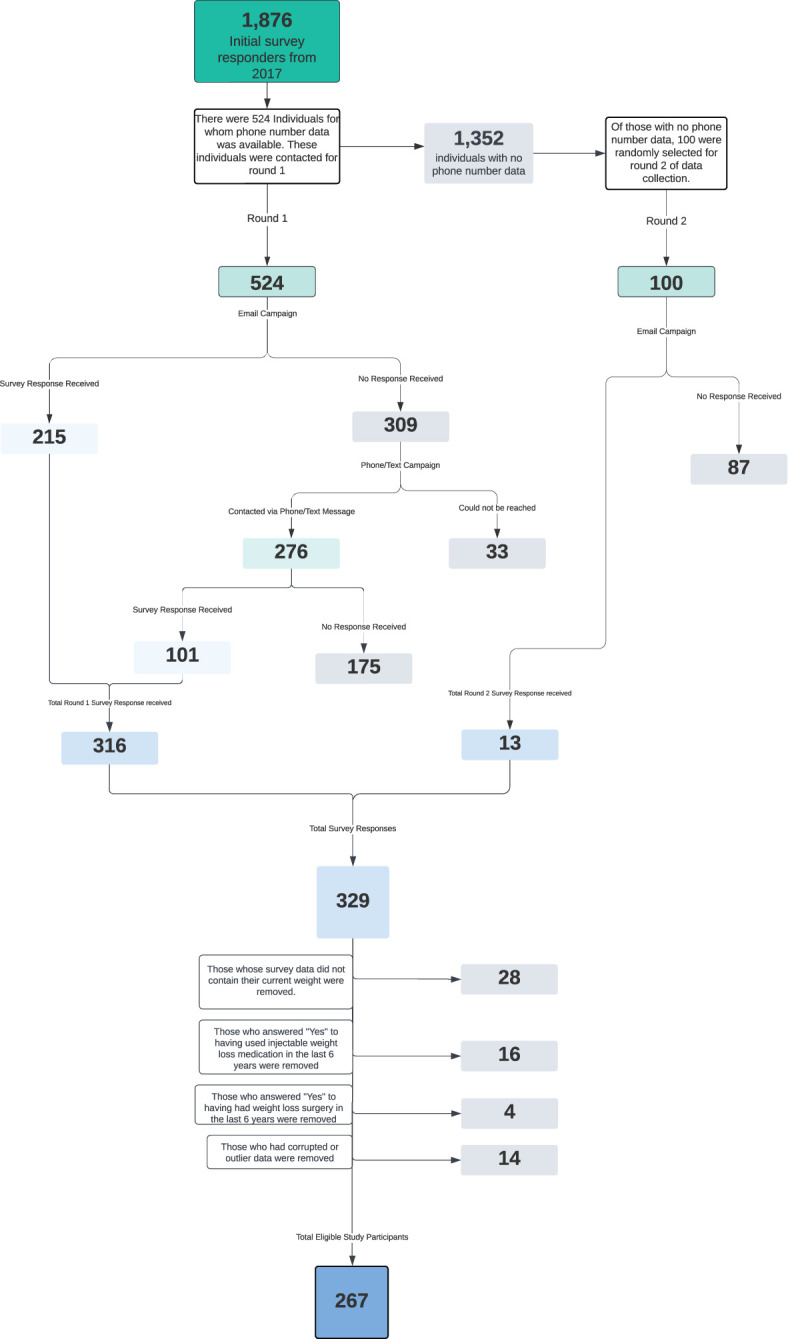
This flowchart illustrates the selection process that resulted in the participants of this study.

Considering the success of the initial email outreach efforts, a decision was made by the research team to return to the remaining 1,352 October 2017 Boot Camp participants and attempt to increase study participation. To avoid selection bias, a random sample of 100 individuals was selected from the 1,352. These 100 individuals received an identical email sequence as the first round of participants. There were 13 survey responses from this email effort representing a 13% response rate. Because we didn’t have phone numbers for these participants, data collection efforts ended there. In total, 329 people filled out surveys, out of 624 people contacted, representing a 52.7% response rate. From there, 28 people were removed because their survey response did not include any current weight data, 16 people were removed for indicating that they had used injectable weight loss medications in the prior six years, 4 people were removed for indicating that they had had bariatric surgery in the prior six years, and 14 people were removed for having either corrupted data or outlier data that was presumed to be erroneous. The following consort diagram reflects the study population selection process (**Figure 1**).

#### 2.2.2 Survey instrument

Both the initial survey that participants responded to at the beginning of their 8-week Boot Camp and the follow-up survey completed six years later included participants’ self-reported demographic information, current weight and height, and weight loss history, which enabled a direct comparison of answers. The current survey included additional questions about participants’ participation in BLE or other weight loss programs over the past six years. Since the initial survey in 2017 did not include the YFAS 2.0, it was not included in the six-year follow-up. The survey was hosted in Alchemer and included a total of 52 questions that employed branching logic (which could potentially reduce the number of questions a participant saw, based on their individual responses). The survey was self-paced, online, and typically required fewer than 30 minutes to complete. Data collection occurred between August 29 and November 3 of 2023.

Participant confidentiality and anonymity were rigorously maintained throughout the study. All collected data were coded and any personally identifiable information was securely stored separately from the research data. Only one investigator had access to the key linking participants to their respective codes. Results presented in this paper are aggregated, ensuring individual anonymity. The study protocol received approval from the BRANY Independent Review Board (IRB) and all participants provided voluntary, informed consent after reading a detailed explanation of the research objectives and privacy practices.

### 2.3 Data analysis

All analyses were performed using Python’s Statsmodels package (version 0.12.2). Continuous outcomes are reported as means with standard deviations and categorical outcomes are reported as frequencies with percentages. For bivariate analyses, one-way ANOVAs were used to compare the differences in weight loss maintenance between groups, and the Pearson’s chi-square test was used to compare the differences among weight tiers. A multiple regression model was performed with the outcome of change between baseline weight and current weight. Independent variables were included in the model if they had a *p* value of < 0.20 in bivariate analyses. Model adequacy and heteroskedasticity were assessed through examination of residuals and model fit statistics. Collinearity among independent variables was assessed by looking at the variance inflation factor. Two-tailed *p* values < 0.05 were considered significant.

## 3 Results

Baseline characteristics of the study participants appear in **Table 1**. The majority of participants were female (97.7%) with an average age of 61.9 years. The majority.

**Table 1:** Baseline demographic characteristics of the study participants.

Statistic	
Gender - N (%)	
Female	259 (97.7%)
Male	6 (2.3%)
Age in years - Mean (SD)	61.9 (9.9)
Race/Ethnicity - N (%)	
American Indian/Alaskan Native/First Nation, Not Hispanic	1 (0.4%)
Asian, Not Hispanic	2 (0.8%)
Black/African American, Not Hispanic	6 (2.3%)
Native Hawaiian/Other Pacific Islander, Not Hispanic	1 (0.4%)
Other - Write In, Hispanic	1 (0.4%)
Other - Write In, Not Hispanic	5 (1.9%)
White, Hispanic	11 (4.2%)
White, Not Hispanic	237 (89.8%)
Height in inches - Mean (SD)	64.5 (3.1)
Baseline (2017) Weight in pounds - Mean (SD)	194.2 (42.6)
Current (2023) Weight in pounds - Mean (SD)	179.7 (42.8)
Baseline (2017) BMI - Mean (SD)	32.9 (6.9)
Current (2023) BMI - Mean (SD)	30.4 (6.9)
Education - N (%)	
High school graduate or GED	11 (4.1%)
Associate's Degree (2-year degree)	19 (7.1%)
Some college	36 (13.5%)
Bachelor's Degree (4-year degree)	74 (27.7%)
Master's degree (including MBA, MPH, ARNP, MPA, MFA)	76 (28.5%)
Some graduate school	27 (10.1%)
Doctoral degree (including MD, PhD, DO, DDS, EdD, JD)	24 (9.0%)
Income - N (%)	
<$40,000	35 (13.5%)
$40,000 - $79,999	60 (23.2%)
$80,000 - $99,999	36 (13.9%)
$100,000 - $149,999	54 (20.8%)
$150,000 or more	74 (28.6%)
Live Alone - N (%)	
Yes	54 (20.5%)
No	209 (79.5%)

of the participants identified as white (89.8%). The mean baseline BMI of 32.9 (SD 6.9) (calculated from their self-reported weight and height) falls above the threshold for type 1 obesity, and a majority of participants (59.1%) had type 1 obesity or above at the start of the program in 2017. Most participants reported some college-level education or above (88.8%) and a household income over $80,000/year (63.3%). Only 20.5% of participants reported living alone. These demographics are not representative of the general population but are typical for populations of US-based commercial weight loss programs (14). Additionally, these characteristics are consistent with those observed in the Bright Line Eating 2-year outcome study published previously (15).

Since completion of the October 2017 Boot Camp, participants followed different paths of engagement and participation in the BLE Membership program and the various online BLE courses. **Table 2** reflects the distribution of levels of engagement among participants. A little under half of participants (43.5%) were current, paying BLE members at the time of the 2023 survey, meaning they had current access to BLE support forums and educational programs, while 56.5% of participants were no longer paying members, meaning that they were either no longer doing BLE, or they were doing it with other tools and support structures or entirely on their own.

**Table 2:** Self-reported length of adherence to the program.

Are they a current member?	
Yes	116 (43.4%)
No	151 (56.6%)
Do they still follow the BLE program?	
Yes	64 (24.1%)
Yes, but not fully	98 (36.8%)
No, but I am thinking about getting back to it	54 (20.3%)
No	50 (18.8%)
Number of years they followed the BLE program (either as member or on their own):	
0	23 (8.6%)
1	32 (11.9%)
2	41 (15.3%)
3	29 (10.8%)
4	33 (12.3%)
5	7 (2.6%)
6	103 (38.4%)

Survey participants were asked whether they were currently following the BLE program (regardless of being paying members) by reporting whether they were adhering to the recommended abstinence rules, the four “Bright Lines” (no flour, no sugar, no eating between meals, and all food weighed and measured).

Roughly one-quarter or 24.1% reported that they were currently following the plan fully (regardless of paid membership status), while 36.8% reported following the plan partially, and 39.1% reported they were no longer following the BLE plan at all (but over half of those said they were “thinking about getting back to it”).

To shed more light on the level of adherence to the program over the span of six years, participants were asked to quantify the number of years from 0–6 that they considered themselves adhering closely to the BLE plan, regardless of their paid membership status. 38.4% of participants reported that they adhered closely to BLE for all six years, while 20.5% of participants reported that they only did BLE for a year or less. **Table 2** presents the self-reported length of adherence to the program reported by all participants.

The self-reported starting weight of all participants reported in October 2017 was available in the BLE research database. The six-year follow-up survey asked for their self-reported current weight and height. Based on the difference, the percent of self-reported body weight gained or lost over those six years was calculated for each participant. Participants were then divided into the following weight-change tiers: weight gain of greater than 5% of starting body weight, weight gain of 0–5%, and weight loss of 0 – <5%, 5 – <10%, 10 – <15%, 15 – <20% and >20%. The distribution of participants among these tiers, both overall and broken down by various interacting variables, is presented in **Table 3**.

**Table 3:** The distribution of participants among weight loss tiers overall and by various interacting variables.

Weight Change								
	Decrease					Increase		p-value
	≥20%	15-<20%	10-<15%	5-<10%	0-<5%	<0 - 5%	>5%	
Total Observations	39 (14.5%)	23 (8.5%)	39 (14.5%)	40 (14.9%)	52 (19.4%)	35 (13%)	40 (14.9%)	
Weight Change (in pounds) - Mean (SD)	-61.6 (21.2)	-33.8 (7.2)	-23.8 (6.7)	-14.9 (3.6)	-4.4 (3.2)	4.1 (2.1)	22.5 (14.7)	<0.001
BMI Category at Baseline								0.01
Normal	0 (0.0%)	2 (6.5%)	2 (6.5%)	6 (19.4%)	11 (35.5%)	4 (12.9%)	6 (19.4%)	
Overweight	6 (7.7%)	3 (3.8%)	15 (19.2%)	9 (11.5%)	18 (23.1%)	12 (15.4%)	15 (19.2%)	
Type 1	14 (18.9%)	12 (16.2%)	12 (16.2%)	10 (13.5%)	8 (10.8%)	11 (14.9%)	7 (9.5%)	
Type 2	7 (16.7%)	5 (11.9%)	5 (11.9%)	8 (19.0%)	9 (21.4%)	2 (4.8%)	6 (14.3%)	
Type 3	12 (27.9%)	1 (2.3%)	5 (11.6%)	7 (16.3%)	6 (14.0%)	6 (14.0%)	6 (14.0%)	
BMI Category Current								<0.001
Normal	17 (28.8%)	5 (8.5%)	10 (16.9%)	8 (13.6%)	13 (22.0%)	4 (6.8%)	2 (3.4%)	
Overweight	14 (14.7%)	14 (14.7%)	18 (18.9%)	9 (9.5%)	18 (18.9%)	11 (11.6%)	11 (11.6%)	
Type 1	5 (9.4%)	3 (5.7%)	6 (11.3%)	12 (22.6%)	9 (17.0%)	10 (18.9%)	8 (15.1%)	
Type 2	2 (6.7%)	1 (3.3%)	3 (10.0%)	7 (23.3%)	7 (23.3%)	4 (13.3%)	6 (20.0%)	
Type 3	1 (3.2%)	0 (0.0%)	2 (6.5%)	4 (12.9%)	5 (16.1%)	6 (19.4%)	13 (41.9%)	
Current Member								<0.001
Yes	27 (23.3%)	13 (11.2%)	21 (18.1%)	17 (14.7%)	19 (16.4%)	12 (10.3%)	7 (6.0%)	
No	12 (7.9%)	10 (6.6%)	18 (11.9%)	22 (14.6%)	33 (21.9%)	23 (15.2%)	33 (21.9%)	
Still Do BLE								<0.001
Yes	28 (43.8%)	10 (15.6%)	14 (21.9%)	5 (7.8%)	2 (3.1%)	4 (6.2%)	1 (1.6%)	
Yes, but not fully	8 (8.2%)	10 (10.2%)	20 (20.4%)	19 (19.4%)	23 (23.5%)	6 (6.1%)	12 (12.2%)	
No, but I am thinking about getting back to it	1 (1.9%)	0 (0.0%)	1 (1.9%)	9 (16.7%)	11 (20.4%)	14 (25.9%)	18 (33.3%)	
No	2 (4.0%)	3 (6.0%)	4 (8.0%)	6 (12.0%)	16 (32.0%)	10 (20.0%)	9 (18.0%)	
Number of years they participated in BLE (either actively or on their own)								<0.001
0	1 (4.3%)	1 (4.3%)	1 (4.3%)	4 (17.4%)	6 (26.1%)	3 (13.0%)	7 (30.4%)	
1	0 (0.0%)	1 (3.1%)	3 (9.4%)	5 (15.6%)	12 (37.5%)	6 (18.8%)	5 (15.6%)	
2	3 (7.3%)	2 (4.9%)	2 (4.9%)	5 (12.2%)	10 (24.4%)	9 (22.0%)	10 (24.4%)	
3	2 (6.9%)	2 (6.9%)	4 (13.8%)	6 (20.7%)	5 (17.2%)	4 (13.8%)	6 (20.7%)	
4	2 (6.1%)	3 (9.1%)	1 (3.0%)	7 (21.2%)	5 (15.2%)	8 (24.2%)	7 (21.2%)	
5	1 (14.3%)	1 (14.3%)	2 (28.6%)	0 (0.0%)	2 (28.6%)	0 (0.0%)	1 (14.3%)	
6	30 (29.1%)	13 (12.6%)	26 (25.2%)	13 (12.6%)	12 (11.7%)	5 (4.9%)	4 (3.9%)	
Participated in Gideon Games								0.07
Yes	17 (17.7%)	13 (13.5%)	18 (18.8%)	11 (11.5%)	15 (15.6%)	12 (12.5%)	10 (10.4%)	
No	22 (12.8%)	10 (5.8%)	21 (12.2%)	29 (16.9%)	37 (21.5%)	23 (13.4%)	30 (17.4%)	
Participated in Accountability Calls								0.01
Yes	21 (23.6%)	10 (11.2%)	13 (14.6%)	16 (18.0%)	12 (13.5%)	9 (10.1%)	8 (9.0%)	
No	18 (10.1%)	13 (7.3%)	26 (14.5%)	24 (13.4%)	40 (22.3%)	26 (14.5%)	32 (17.9%)	
Age								0.03
<50 years	1 (3.0%)	2 (6.1%)	4 (12.1%)	6 (18.2%)	5 (15.2%)	5 (15.2%)	10 (30.3%)	
50–59 years	6 (8.6%)	6 (8.6%)	6 (8.6%)	8 (11.4%)	16 (22.9%)	13 (18.6%)	15 (21.4%)	
60-69 years	22 (21.4%)	9 (8.7%)	15 (14.6%)	14 (13.6%)	22 (21.4%)	10 (9.7%)	11 (10.7%)	
>69 years	10 (16.7%)	6 (10.0%)	14 (23.3%)	11 (18.3%)	8 (13.3%)	7 (11.7%)	4 (6.7%)	

39 participants (14.5%) reported a weight that represented a loss of 20% or more of their body weight six years after beginning the Boot Camp, 23 (8.5%) reported a weight that represented a loss 15–20% of their body weight, 39 (14.5%) reported a weight that represented a loss 10–15%, and 40 (14.9%) reported a weight that represented a loss of 5–10%. Some participants reported weighing the same or more than when the Boot Camp began. Specifically, 35 participants (13%) reported a weight that was 0–5% above their baseline reported weight and 40 (14.9%) reported a weight that was more than 5% above their reported baseline weight.

**Figure 2:**
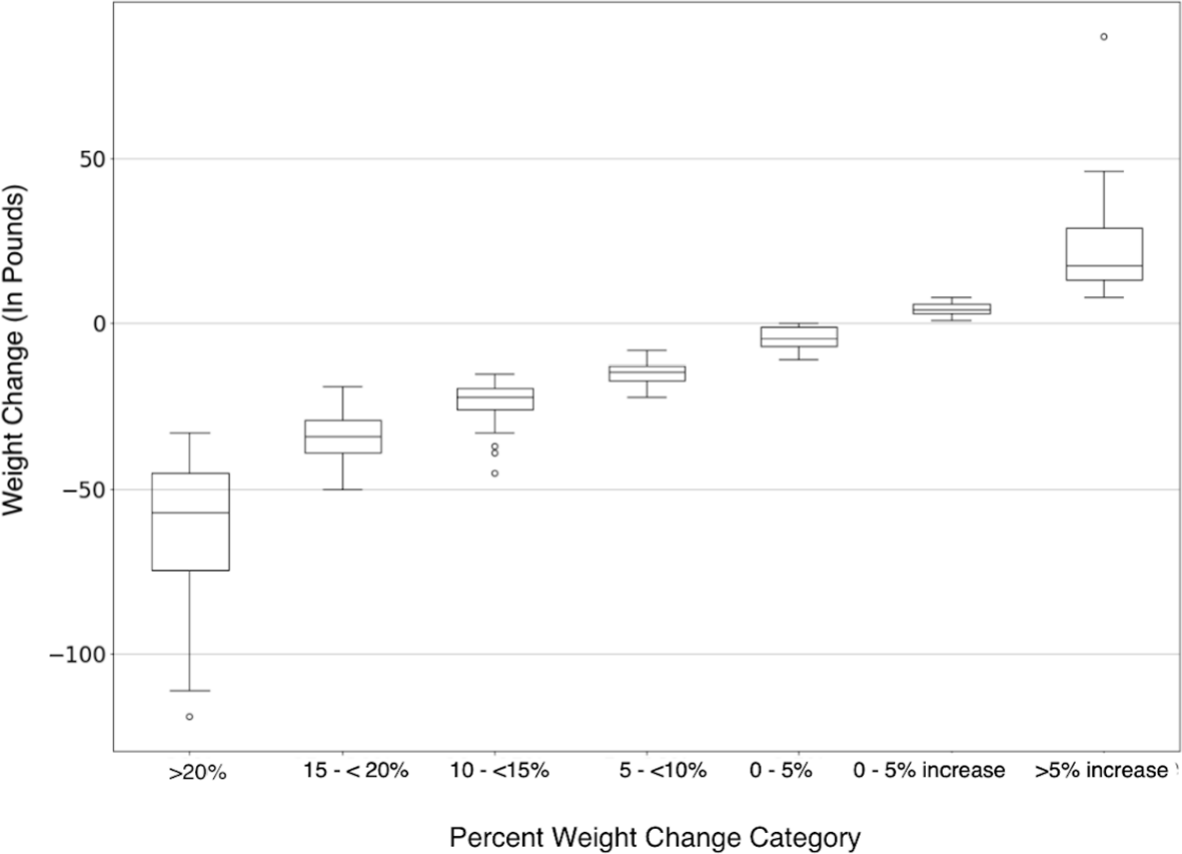
Magnitude of weight change in pounds is on the y-axis, each percent weight loss category is represented on the x-axis. Negative numbers indicate weight decreased from baseline (weight loss) and positive numbers indicate weight increased from baseline (weight gain).

**Figure 2** shows the mean reported weight loss for each tier of percent body weight lost. The mean reported weight loss for the group that reported losing 20% or more of their body weight was 61.6 lbs. (SD 21.2). The mean reported weight loss for all participants was 14.5 lbs. Of those participants who reported gaining more than 5% of their body weight, the mean weight gain was 22.5 lbs. (SD 14.7). The difference between the weight loss means for the different tiers was highly statistically significant, *p*<0.001.

When examining the relationship between membership status in the BLE program and reported weight loss success, we found a highly statistically significant association (*p*<0.001) that was maintained across all weight loss tiers and was reversed in the weight gain tiers. In the >20% reported weight loss tier, 69% of the people were active members of BLE and 31% were non-members. Conversely, 82.5% of the people who reported gaining more than 5% of their body weight were not members of BLE while only 17.5% were current members.

Additionally, there was a very strong association between weight loss tier and whether or not the participant self-reported adhering to the BLE program (*p*<0.001). In the >20% reported weight loss tier, 72% of people reported following the BLE plan fully. Conversely, of the 40 participants who reported gaining more than 5% of their body weight in six years, only 1 participant (2.5%) reported adhering to the program fully, while most participants (67.5%) reported not following the BLE program at all. In this analysis, a strong “dose response” to program adherence was observed, with people not following the BLE program reporting losing very little weight or gaining weight, people somewhat following the program report modest results, and people adhering fully, report achieving, and then maintaining, significant weight loss, on average.

**Figure 3:**
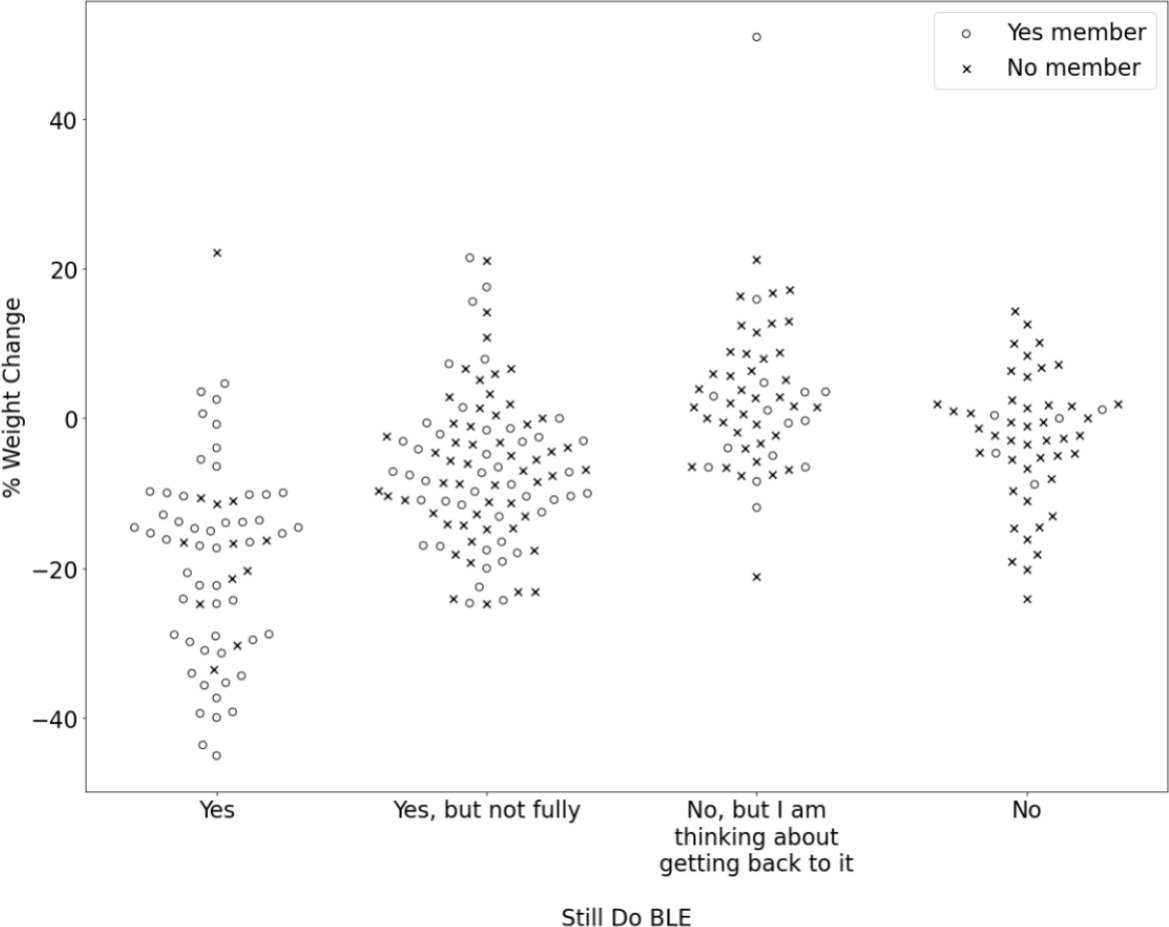
Percent weight change is represented on the y-axis and participants' response to the question, "Are you still following the Bright Line Eating plan?" is represented on the x- axis. An open circle represents that the participant indicated "Yes" to the question, "Are you currently a member of Bright Line Eating?" and an "x" represents that the participantindicated "No.".

**Figure 3** depicts the relationships among level of adherence to the BLE program, paying membership status, and the percent of reported body weight lost (or gained) after six years. Those same relationships are represented numerically in **Table 4**.

**Table 4:** The relationships among the level of adherence to the BLE program, paying membership status, and percent self-reported body weight change.

Average % Weight Change		
Entire cohort		-6.87%
.5	Fully Following BLE	Not Fully Following BLE
Current members	-13.91%	1.38%
Non-Members	-7.88%	0.05%

Along the same lines, when we examined the relationship between the number of years people self-reported participating in the program (either as active members or on their own) and the weight loss tiers (**Table 2**), we found that the number of years was associated with reported weight loss success in a “dose-response” manner. In the highest weight loss tier, 80% of people who reported losing >20% of their body weight were those who reported participating in the program for 5–6 years. In contrast, 88% of people who reported gaining more than 5% of their body weight reported following the program for 0–4 years.

Participants were asked about participation in specific BLE activities in order to understand the association between these activities and success in the program. The findings suggest that peer-to-peer and community support are correlated with sustained weight loss success. Regular self-reported participation in at least some of the “Accountability Calls” with coaching and daily celebrations, hosted for 30 minutes every morning and evening live on Zoom by BLE coaches, was statistically significantly associated with long-term weight loss success (*p*=0.03). Across the weight loss tiers, those reporting participating were more successful than those not participating. This relationship was reversed in the reported weight gain tiers, where the vast majority of participants reported that they did not participate in the calls. Age above 60 was statistically significantly associated with greater reported weight loss success, *p*=0.03. None of the remaining participant demographic variables–level of education, income, race, and whether or not they lived alone–had a statistically significant relationship to weight loss outcomes.

A multiple regression model was performed with the outcome of change in weight between baseline and six-year-follow-up. Independent variables were included in the multiple regression model if they had a *p* value of < 0.20 in bivariate analyses. While controlling for other variables, the only variable that was highly statistically significant was whether the participant followed the BLE program completely, partially, or not at all. Following the BLE program completely was highly statistically significantly associated with reported weight loss success, *p*<0.001.

## 4 Discussion

The results of this six-year follow-up study support the long-term efficacy of an abstinence-based, food addiction recovery approach to sustained weight loss maintenance. Participants who remained actively engaged with the program and continued to follow the prescribed abstinence plan reported achieving clinically significant weight loss at levels.

In the current study, 89% of participants who were still adhering to the abstinence-based food addiction recovery program reported a greater than 5% weight loss, six years later. And 44% of participants who were still doing the program fully reported a greater than 20% weight loss. In this >20% tier, the mean reported weight loss was 61.6 lbs. (SD 21.2). And among ALL participants, including those who weren’t following the program anymore and those who participated in BLE for just 0–1 years, the average weight loss reported, six years later, was 6.9% or 14.5 pounds, showing that the eight weeks of exposure to an abstinence-based food plan and education about food addiction might have provided a measure of “inoculation” against the weight gain that is observed over time in the general population. The study’s self-reported data also did not indicate a propensity for significant weight rebound in the majority of the participants.

Even though not assessed directly in this cohort, based on YFAS 2.0 assessment, a majority (64.4%) of enrollees in a 2024 BLE Boot Camp qualified for a diagnosis of food addiction (16). Of those, a majority qualified for a diagnosis of severe food addiction, having both clinically significant impairment or distress and six or more symptoms of food addiction. As the demographic, recruiting methods, and other characteristics of the Boot Camp cohorts have been stable over time, it is reasonable to extrapolate the presence of a high prevalence of food addiction in the current study cohort as well. Thus, it is reasonable to conclude that an abstinence-based food plan coupled with a food addiction recovery program is an effective long-term weight management approach both for people diagnosed with food addiction and for those in the general population.

Recently, there has been intense focus on the significant weight loss that the single receptor agonist GLP-1s and now the double and triple (GLP-1-GIP and GLP-1-GIP-GCG) receptor agonists have been achieving (17). Semaglutide, the most common GLP-1 for weight loss, has been shown to produce 15% weight loss at two years (18). Some of the newest double and triple agonists are achieving ~25% body weight loss in early trials, with most follow-ups still under two years (16). The exact mechanism of action of these medications is still not totally known and is likely multifactorial, but appetite reduction is certainly one of the major mechanisms by which these medications exert their weight loss effect (19).

Another proposed mechanism is that these medications reduce food cravings by impacting the central nervous system and in particular areas in the brain central to addiction (20). This proposed mechanism has led to trials testing the impact of these medications on other addictions including alcohol, cocaine, nicotine, stimulants and even gambling (21). It is interesting that individuals who reported adhering fully to the BLE program, which has food addiction treatment at its core, had reported weight loss similar to those achieved by individuals taking a GLP-1 receptor agonist. This might offer support to the assertion that addiction is a central cause of obesity in many individuals. We hypothesize that one of the reasons that BLE participants reported the levels of sustained weight loss that they did and that receptor agonist GLP-1s are so highly effective is that both address food addiction which is one of the central, underlying causes of obesity. BLE does so behaviorally, and the medications do so pharmacologically. The current study sheds light on an approach to weight loss that does not share the side effects (22), costs (23), nor required long-term continuation of GLP-1 medications (24). Future research will be essential to determine whether BLE may offer an alternative to weight loss medications in patients who are unable to afford or tolerate them, or as a post-medication intervention to help sustain the weight loss achieved after their discontinuation.

There has been suggestion in the literature (25, 26) that treatment of food addiction with an abstinence-based approach may actually increase cravings and thereby result in weight gain. Because of the limitations of our data, we are not able to conclusively state whether this is the case, although the data in our study does indicate that over a six-year period the participants most likely to report weight gain were those reporting that they were not adhering to the program. There was a small percentage (~7%) of participants who reported adhering to the program who reported weight gain despite active participation in the program. Most of these participants reported gaining less than 5% of their body weight. And the cohort overall, including both adherents and non-adherents, reported weight loss (6.9% of body weight) at six years. Additional research would be needed to further elucidate this concern.

Our current study had several limitations. The fact that we did not have objective baseline or follow up measured weights is a central limitation of our study. This is a challenge that other non-clinical, not-in-person programs face. Research shows that there is a moderate under-estimation of starting weight that is impacted by demographic factors such as age, ethnicity and education levels. The correlation between self-reported and measured weights was 0·97. In one study, on average, women under-reported their weight by about 2 lbs. (0.91 kg) (27).

Some studies have chosen to apply a correction factor to participants’ reported weight. We chose not to apply a correction factor because our study included a relatively demographically homogeneous group and included both adherers and non-adherers to the program. We assumed that both groups would likely underestimate their starting weight and current weight similarly and that we might add additional bias and complexity by applying a correction equation (28).

A second limitation is the risk of selection bias because individuals who were more successful might be more likely to respond to the follow-up survey. Interestingly, more individuals who were no longer paying members of the program responded to the survey than did those who were active current paying members (specifically, 56.5% of respondents were non-members and 43.5% were current, paying BLE members at the time of the six-year survey). Many people who were no longer members and no longer adhering to the abstinence-based food plan responded to the survey. However, selection bias is still a limitation.

A third limitation is that, since this was an observational study, we are unable to ascribe causality to our findings. We can only conclude that participating in an abstinence-based, food addiction recovery program predicts (rather than causes) long-term weight loss maintenance.

In addition, we did not have formal YFAS 2.0 scores specific to this cohort which prevents us from definitively assessing the percentage of people in the cohort who had food addiction, either at baseline or at follow-up.

Finally, the study sample was very homogeneous. There were few men in the sample, few people of color, and few socioeconomically challenged participants. Overall, the homogeneity of the study’s sample population in terms of sex, age, race/ethnicity, geographic location, and socioeconomic status limits the ability to generalize these results to a broader population.

### 4.1 Conclusion

The findings of this six-year follow-up of a cohort of people who participated in an eight-week weight loss program in 2017 suggest that an abstinence-based food plan coupled with a food addiction recovery program may be an effective long-term weight management approach, particularly for individuals with food addiction symptoms. However, further controlled studies are needed to confirm causality and generalizability to broader populations.

## Data Availability

The raw data supporting the conclusions of this article will be made available by the authors, without undue reservation.
